# Computational tissue staining of non-linear multimodal imaging using supervised and unsupervised deep learning

**DOI:** 10.1364/BOE.415962

**Published:** 2021-03-23

**Authors:** Pranita Pradhan, Tobias Meyer, Michael Vieth, Andreas Stallmach, Maximilian Waldner, Michael Schmitt, Juergen Popp, Thomas Bocklitz

**Affiliations:** 1 Institute of Physical Chemistry and Abbe Center of Photonics, Friedrich-Schiller-University, Jena, Germany; 2 Leibniz Institute of Photonic Technology, Member of Leibniz Health Technologies Jena, Germany; 3 Institute of Pathology, Klinikum Bayreuth, Bayreuth, Germany; 4 Department of Internal Medicine IV (Gastroenterology, Hepatology, and Infectious Diseases), Jena University Hospital, Jena, Germany; 5 Erlangen Graduate School in Advanced Optical Technologies (SAOT), Friedrich-Alexander University of Erlangen-Nuremberg, 91052 Erlangen, Germany; 6 Medical Department 1, Friedrich-Alexander University of Erlangen-Nuremberg, Erlangen, Germany

## Abstract

Hematoxylin and Eosin (H&E) staining is the ’gold-standard’ method in histopathology. However, standard H&E staining of high-quality tissue sections requires long sample preparation times including sample embedding, which restricts its application for ’real-time’ disease diagnosis. Due to this reason, a label-free alternative technique like non-linear multimodal (NLM) imaging, which is the combination of three non-linear optical modalities including coherent anti-Stokes Raman scattering, two-photon excitation fluorescence and second-harmonic generation, is proposed in this work. To correlate the information of the NLM images with H&E images, this work proposes computational staining of NLM images using deep learning models in a supervised and an unsupervised approach. In the supervised and the unsupervised approach, conditional generative adversarial networks (CGANs) and cycle conditional generative adversarial networks (cycle CGANs) are used, respectively. Both CGAN and cycle CGAN models generate pseudo H&E images, which are quantitatively analyzed based on mean squared error, structure similarity index and color shading similarity index. The mean of the three metrics calculated for the computationally generated H&E images indicate significant performance. Thus, utilizing CGAN and cycle CGAN models for computational staining is beneficial for diagnostic applications without performing a laboratory-based staining procedure. To the author’s best knowledge, it is the first time that NLM images are computationally stained to H&E images using GANs in an unsupervised manner.

## 1. Introduction

Conventional staining technique like histopathological (H&E) staining is the ’gold-standard’ technique for tissue diagnostics. High quality H&E staining requires an embedding of the sample in paraffin to generate FFPE sections, which is time-consuming. Due to the time requirement, conventional H&E staining (using FFPE material) cannot be used for real-time disease diagnosis like in a cryosection setting, where low quality cryosections are stained. Additionally, this technique can only show limited biomolecular information. If bio-molecular information is needed for diagnostics, it must be acquired using molecular imaging techniques. Thus, in our work, one of the molecular imaging techniques like non-linear multimodal (NLM) imaging is used, which can complement the ’gold-standard’ histopathological staining technique. The NLM imaging used here is based on cryosection material which means that the sample is not embedded in FFPE and thus have a time advantage. In that way, biomolecular information can be extracted, and this technique can be applied for real-time disease diagnosis [1,2]. As NLM images used in our study are based on cryo material that were followed by H&E staining, we do not show images of H&E stained FFPE sections (instead histopathologically stained H&E images).

The NLM imaging presented here is a combination of three non-linear optical modalities, namely coherent anti-Stokes Raman scattering (CARS) microscopy, two-photon excitation fluorescence (TPEF) microscopy and second-harmonic generation (SHG) microscopy. These three modalities highlight the distribution of biomolecules like collagen, NADH, proteins and lipids [2,3]. Furthermore, NLM imaging is label-free and provide highly resolved images of biological tissues. The non-destructive nature of NLM imaging is suitable for *in vivo* studies. Due to these properties and the fact that NLM imaging provides morphological and functional information of a tissue sample, this imaging technique is beneficial for tissue imaging and other biomedical applications [3] like investigations of skin diseases [4–6], diagnostics of head-neck cancer [7,8], classification of brain tumors [9], and characterization of inflammatory bowel disease samples [10].

Despite the ever-increasing use of NLM imaging, its establishment in clinics is not achieved until now. In situations where cryo sections are analyzed, NLM would be a great technique, because the computational staining is of higher quality as H&E stains in a cryosection analysis. The NLM images have a higher resolution compared to the histopathologically stained H&E images and exhibit color contrast which is unfamiliar to physicians. For diagnostics, physicians tend to screen histopathologically stained H&E images and then zoom into suspicious regions for diagnostics. This is problematic as the contrast of NLM images is different from corresponding histopathologically stained H&E images, and NLM images can be zoomed to higher tissue levels. Thus, interpretation of NLM images with its corresponding histopathologically stained H&E images is challenging. To interpret the NLM images and link it to standard histopathologically stained H&E images, a parallel tissue section and afterwards the cryosection are stained with conventional staining procedures. Subsequently, the stained images are compared with the corresponding NLM images. This comparison is laborious, which reduces the advantage of NLM imaging. Therefore, comparison of histopathologically stained H&E image and NLM image requires an automatic translation of both images. Furthermore, an automatic translation of different modalities to H&E stained image can aid intraoperative histopathologic diagnosis and efficient decision-making during surgery [11]. Such an automatic model can also generate awareness and trust in the new NLM imaging technique.

**Fig. 1.**
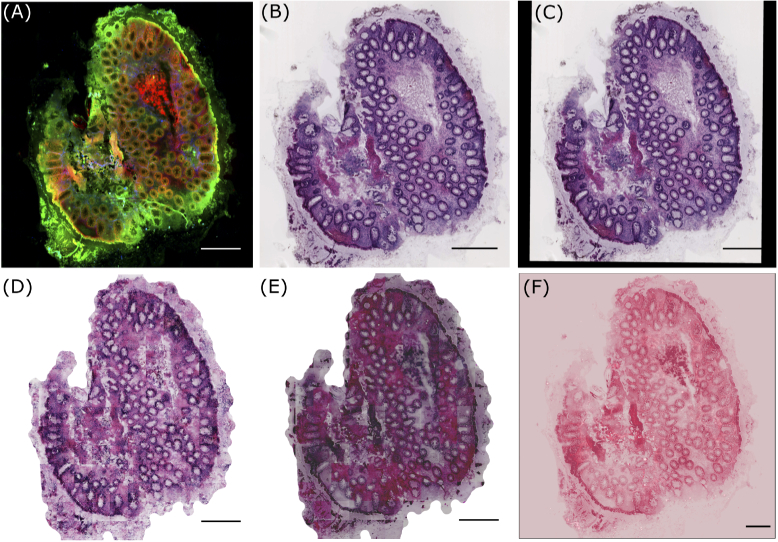
(A) shows a pre-processed NLM image with CARS, TPEF and SHG as the red, green and blue channel, respectively, (B) visualizes histopathologically stained H&E image (or unregistered H&E image) used for unsupervised pseudo-stain H&E model, (C) depicts a registered H&E image used for supervised pseudo-stain H&E model. The image in (C) shows the registration effect, which is filled with zeros. The images in (D), (E) and (F) are computationally stained H&E images with the supervised, unsupervised approach and method used in reference [12], respectively. All images are downscaled to 20% of the original size for clarity. The scale bar in all images represents 100 μm.

In this context, researchers in 2016 performed the modality transfer of NLM images to histopathologically stained H&E images by image analysis and machine learning methods [12]. Although their work showed comparable results (see Fig. 1), the approach had two limitations. Foremost, the colors in the computationally stained H&E images were different compared to the original histopathologically stained H&E images. Secondly, their work trained a machine learning model that required a corresponding pair of NLM images and histopathologically stained H&E images. This training procedure is time-consuming as the histopathologically stained H&E image of the same tissue section must be prepared and registered to the coordinate space of the NLM image before constructing the machine learning model. In most cases, the multimodal image registration is a difficult task due to tissue alterations that occur during the staining procedure.

In contrast, our work presents an improvement of the work of Bocklitz et al., 2016 in terms of the staining results and the required manual effort for modality transfer. This was achieved by utilizing deep learning models instead of conventional machine learning methods. Briefly, deep learning models, specifically generative adversarial networks [13], were utilized to translate NLM images into computationally stained H&E images. This work was performed in a supervised and an unsupervised approach, where a paired [14] and an unpaired image translation [15] of the NLM image was performed, respectively. The supervised approach or paired image translation required a corresponding pair of images measured with the two modalities (NLM imaging and H&E staining), while the unsupervised approach did not require paired images of the two modalities. Like the previous work of Bocklitz et al. 2016, the supervised approach has the limitation of registering the histopathologically stained H&E images to the corresponding NLM images. On the other hand, the unsupervised approach does not require the image registration of the two modalities. Moreover, the unsupervised approach offer additional advantages like the artificial generation of images from both modalities, the translation of images from multiple modalities and minimal requirement of stained images.

The supervised and unsupervised approach utilized a conditional generative adversarial network (CGAN) [16] and a cycle conditional generative adversarial network (cycle CGAN) [14], respectively. CGANs are commonly used in computer vision tasks for translating images [14], but they were never used to translate an NLM image to a H&E stained image. Common applications of CGAN in computer vision are the transformation of photographs acquired in daylight into photographs of night scenes, or the transfer of horse images into images of zebras. Likewise, its application in the biomedical and optical field is gaining popularity [17–23]. Recent works transformed auto-fluorescence images [24] or hyperspectral images [25] into histopathologically stained H&E images using CGANs. A similar approach was performed for translating quantitative phase imaging into three different stains, namely H&E stain, Jone’s stain, and Masson’s trichrome stain [26]. CGANs were also employed to increase the spatial resolution [27,28] and remove speckle noise from optical microscopic images. Similarly, the cycle CGAN were utilized to stain a H&E stained image into an immunohistochemically (IHC) stained image. The generated IHC image was used to reconstruct the original H&E stained image [29]. As mentioned earlier, unpaired image translation is advantageous as co-registration of images from different modalities is not needed, but the model for the unpaired image translation needs to be more complex as compared to the model for paired image translation.

Our work is different from the state-of-the-art methods because it is the first time that NLM images were used for an unsupervised transfer to histopathologically stained H&E images. While performing the unsupervised modality transfer, the corresponding difficulties were tackled. First of all, the different contrast between the two modalities makes the image translation task complicated. Furthermore, the NLM images used in this work are measured from tissue of patients with different disease severity (namely Inflammatory bowel disease), which is reflected in the alterations of the tissue structure and changes in the pixel contrast [30]. The availability of NLM images is limited, which is problematic because the training of adversarial networks requires large datasets. Lastly, we evaluated the modelling quantitatively by considering perceptual or texture information and color information. Overall, this work is an improvement of the state-of-the-art method presented by Bocklitz et al. in 2016 [12], based on paired and unpaired image translation of NLM images into histopathologically stained H&E images.

## 2. Material and methods

### 2.1 Dataset

The dataset used in this work is published elsewhere [30]. Briefly, it consists of tissue samples from biopsies of patients with Crohn’s disease, ulcerative colitis or infectious colitis obtained during colonoscopy or surgical resections. The dataset has 19 pairs of NLM images and histopathologically stained H&E images (see Fig. 1). The NLM image is an RGB image where each channel represents one of the three non-linear optical modalities. Precisely, the CARS signal, the TPEF signal, and the SHG signal form the red, green and blue channel of the RGB image, respectively. The spatial (pixel) resolution of the NLM image is 0.227 μm/pixel (see Fig. 1(A)). For the histopathologically stained H&E images, the corresponding tissue sections were stained in the pathology department. The (digital) histopathologically stained H&E images in the form of slide scanner files were extracted using Aperio Image scope software with a spatial resolution approximately equal to the NLM image. The spatial resolution of the extracted histopathologically stained H&E image is 0.219 μm/pixel (see Fig. 1(B)). This spatial resolution setting was favourable for image registration step. The corresponding pairs of NLM and histopathologically stained H&E images were used to construct a “pseudo-stain H&E model” based on the conditional generative adversarial networks in a supervised and an unsupervised approach. The pseudo-stain H&E model was trained using 13 image pairs and tested on six image pairs. For building the pseudo-stain H&E model, both images were pre-processed, and the histopathologically stained H&E image was registered to the NLM image only for the supervised approach.

### 2.2 Image pre-processing of the histopathologically stained H&E image

**Fig. 2.**
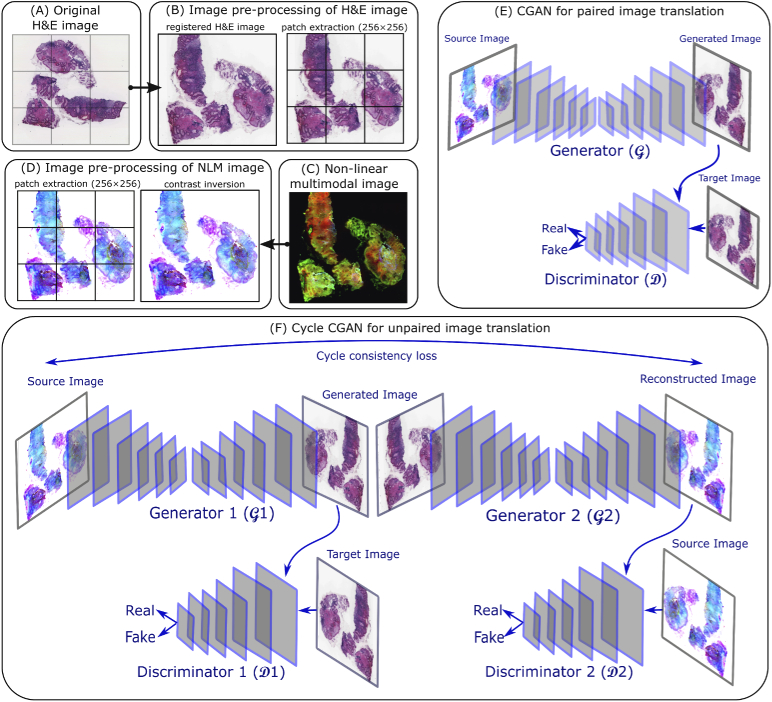
(A) is a histopathologically stained H&E image, (B) shows the image pre-processing of a histopathologically stained H&E image including image registration and patch extraction of size 256×256, (C) is a corresponding NLM image, (D) visualizes the contrast inversion of the NLM image followed by patch extraction of size 256×256, (E) shows a CGAN model for paired image translation which utilizes the registered histopathologically stained H&E images and contrast inverted NLM images, (F) depicts a cycle CGAN model for unpaired image translation using unregistered histopathologically stained H&E images and contrast inverted NLM images.

The histopathologically stained H&E image was registered to the coordinate space of the corresponding NLM image using the Image processing toolbox in Matlab 2018a. For the image registration purpose, the NLM and histopathologically stained H&E images were converted to grayscale, followed by contrast inversion of the histopathologically stained H&E image. The contrast inversion was achieved by subtracting the pixel values in each channel of the H&E image by 255. The contrast inversion of histopathologically stained H&E image was performed only for image registration purpose (not for model training). The inverted histopathologically stained H&E image (grayscale) was used as a moving image, and the corresponding NLM image (grayscale) was used as a fixed image. Subsequently, a multimodal image registration [31] based on the mutual information metric was performed using the NLM and the histopathologically stained H&E images. The registered histopathologically stained H&E image (see Fig. 1(C)) was used for the supervised approach or paired image translation, while the unregistered histopathologically stained H&E image (see Fig. 1(B)) was utilized for the unsupervised approach or unpaired image translation. Further, patches of size 256×256 were extracted from the registered and the unregistered histopathologically stained H&E image. All the histopathologically stained H&E patches were scaled in the range [−1,1] before model training. The patches from the registered histopathologically stained H&E image were used to train the CGAN model, while the patches from the unregistered histopathologically stained H&E image were used to train the cycle CGAN model (see Fig. 2(A) and Fig. 2(B)).

### 2.3 Image pre-processing of the non-linear multimodal image

The data acquisition and pre-processing of NLM images were similar to Chernavskaia et al., 2016 [30]. Briefly, the pre-processing steps included median filtering, downsampling by a factor of 4, correcting the uneven illumination and adjusting the contrast of the NLM images. A pre-processed NLM image is shown in Fig. 1(A). Subsequently, the contrast of NLM images was inverted by subtracting the pixel values by 255. Contrast-inversion of NLM images was performed only for GAN model training. Further, patches of size 256×256 were extracted from the “contrast-inverted” NLM image (see Fig. 2(C) and Fig. 2(D)). These patches were filtered separately for the supervised and unsupervised approach. For the supervised approach or the CGAN model, the pair of NLM and histopathologically stained H&E patch showing registration artefact were removed. The registration artefacts were seen at the borders of the registered histopathologically stained H&E image, which were filled with zero values during registration (see Fig. 1(C)). For the unsupervised method or the cycle CGAN model, the NLM and histopathologically stained H&E patches belonging to the background region were removed using the homogeneity factor [32], i.e. the patches with homogeneity factor greater than 60% were removed [32]. Similar to the H&E patches, all the selected NLM patches were normalized in the range [−1,1] before model training.

### 2.4 Conditional generative adversarial network

**Fig. 3.**
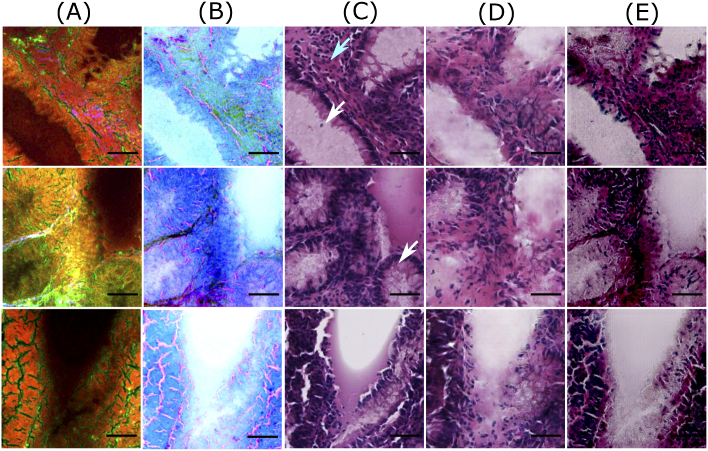
Good quality predictions of patches from the training dataset. Columns (A) and (B) visualize NLM patches and contrast inverted NLM patches, column (C) shows histopathologically stained H&E patch, and columns (D) and (E) visualize computationally stained H&E patch generated by the Pix2Pix model and the cycle CGAN model, respectively. The scale bar represents 50 μm. For all patches in column C, the region within the crypts (pointed by white arrows) is light or pale pink, whereas the epithelial layer or mucosa region outlining the crypts appears dark purple. Similar colors with few variations are observed in the computationally stained H&E patches generated by the Pix2Pix (column D) and the cycle CGAN (column E) model. Also, the crypt structures are efficiently generated in the computationally stained H&E patches by both models. It is also observed that nucleus (pointed by cyan arrow) are not generated by the cycle CGAN model (column E).

The conditional generative adversarial network (CGAN) used in this work was inspired by the Pix2Pix model developed by Isola et al., 2017 [14]. The Pix2Pix model comprised of a generator (G) and a discriminator (D) (see Fig. 2(E)). The generator with an autoencoder architecture [33] transforms a contrast-inverted NLM patch ($x_{m}$) to a computationally stained H&E patch ($z_{generated}=G(x_{m})$) which looked visually similar to the histopathologically stained H&E patch ($z_{target}$). The input to the generator was a pre-processed NLM patch (see column B in Fig. 3) and a target or histopathologically stained H&E patch (see column C in Fig. 3). The computationally stained H&E patch, i.e. output of the generator, (see column D in Fig. 3) was evaluated by calculating mean absolute error with the target histopathologically stained H&E patch and was optimized to be minimal. The discriminator model was trained to predict the plausibility of the computationally stained H&E patch ($z_{generated}$). In simpler words, the discriminator model was trained to predict if the computationally stained H&E patch was ’fake’ (i.e. not belonging to histopathologically stained H&E patches) or ’real’ (i.e. belonging to the original dataset of histopathologically stained H&E patches). The details of the generator and discriminator networks are elaborated below.

The generator network was inspired by the U-Net model [34] which is an autoencoder. The autoencoder model had eight blocks in the encoder and the decoder part. Each block of the encoder utilized convolution layer, batch normalization layer and Leaky ReLU activation layer. The last layer of the encoder was a bottleneck layer without batch normalization layer. The eight encoder blocks comprised of 64, 128, 256, 512, 512, 512, 512 and 512 filters, respectively. On the other hand, each decoder block comprised of a convolution layer, batch normalization layer, dropout layer with a 50% dropout rate and ReLU activation layer. Like the encoder network, the first layer of the decoder did not use a batch normalization layer. The layers in eight decoder blocks comprised of 512, 1024, 1024, 1024, 1024, 512, 256 and 128 filters, respectively (after concatenation from the encoder). All the convolutional layers in the encoder and the decoder blocks used a kernel size of 4 and stride size of 2. The encoder and the decoder models were linked through ’skip-connections’ similar to the U-Net architecture. The output layer of the generator was a single convolutional layer with three channels and tanh activation function. The output of the generator was a computationally stained H&E patch ($z_{generated}=G(x_{m})$) which was one part of the discriminator network’s input.

The discriminator network was a standard convolutional neural network with input as computationally stained H&E patch ($z_{generated}$) and histopathologically stained H&E patch ($z_{target}$) of size 256×256. The architecture of the discriminator network was inspired by the ’PatchGAN’ discriminator given in Ref. [14]. The basic idea of the PatchGAN discriminator model is to classify an N×N region in the M×M input image (N<M) as ’real’ or ’fake’, instead of classifying the whole M×M input image as ’real’ or ’fake’. In our case, M=256 and N=70 i.e. a 70×70 region in the 256×256 computationally stained H&E patch was classified as ’real’ or ’fake’. The 70×70 region is termed as the ’receptive field’. The output of the discriminator model was a map with 16×16 values scaled using a sigmoid activation function. In other words, each value in the 16×16 sigmoid activation map corresponded to the probability of the 70×70 region in the input patch being ’real’ (1.0) or ’fake’ (0.0). These values were combined to achieve a single probability value, which corresponded to the probability of the entire input patch being ’real’ or ’fake’. The layers of the PatchGAN discriminator model were adjusted to maintain the receptive field size to 70×70. Specifically, the layers of the PatchGAN discriminator model used 64, 128, 256 and 512 filters respectively, and Leaky ReLU activation function with slope 0.2. The configuration of the Leaky ReLU activation function, kernel size and stride size were the same for both the generator and discriminator networks.

Before training of generator and discriminator networks, the weights of both networks were initialized using random Gaussian numbers with a standard deviation of 0.02. During the training phase, the weights of the discriminator model were updated by a set of histopathologically stained H&E patches ($z_{target}$) and computationally stained H&E patches ($z_{generated}$), and calculating the discriminator loss

(1) $$L_{D}=D{\left(z_{generated}\right)}^{2}+{\left(1-D(z_{target})\right)}^{2}.$$

When the discriminator network is better than the generator network, i.e. $D(z_{target})=1$ and $D(z_{generated})=0$, it is able to identify all the computationally stained H&E patches as ’fake’. To avoid the discriminator network to become better than the generator network, the training process of the discriminator network was slowed down by weighting the discriminator loss $L_{D}$ by 50% for each model update [35]. The ideal case is to converge the discriminator loss to 0.5 and the generator to create H&E patches exactly similar to the target histopathologically stained H&E patches. On the other hand, the weights of the generator network were updated by calculating the mean absolute error between $z_{generated}$ and $z_{target}$. Additionally, the weights of the generator network were updated through the adversarial loss obtained from the discriminator network. Thus, the total loss of the generator network $L_{G}$ is given by

(2) $$L_{G}=\lambda \text{MAE}(z_{generated},z_{target})+{\left(1-D(z_{generated})\right)}^{2},$$

where the mean absolute error (MAE) was weighted by a hyperparameter λ. In our case, λ was set to 10. The weights of the generator and discriminator networks were updated separately to avoid misleading updates. Furthermore, both networks were trained using the Adam optimizer [36] with learning rate and β set to 0.0002 and 0.5, respectively.

### 2.5 Cycle conditional generative adversarial networks

The cycle CGAN model is an extension of the conditional generative adversarial network which does not require paired images for the image translation task [15]. The cycle CGAN model involved simultaneous training of two generators $\left(G_{1},G_{2}\right)$ and two discriminators $\left(D_{1},D_{2}\right)$ (see Fig. 2(F)). The first generator $G_{1}$ utilized a contrast-inverted NLM patch $\left(x_{m}\right)$ (see Fig. 3, column B) as input and generated an H&E stained patch as output $\left(z_{generated}=G_{1}(x_{m})\right)$ (see Fig. 3, column E). The second generator utilized the computationally stained H&E patch (i.e. the output of the generator 1, $z_{generated}$) as input and reconstructed it to the original NLM patch (similar to the input of the generator 1, ${\tilde{x}}_{m}=G_{2}(z_{generated})$). The output of the second generator $\left({\tilde{x}}_{m}\right)$ was optimized to be visually similar to the input of the first generator $\left(x_{m}\right)$ and was regularized by calculating the (forward) cycle consistency loss $L_{\left(cyc_{f}\right)}$. In similar fashion, backward cycle consistency loss $L_{\left(cyc_{b}\right)}$ regulated the second generator. Additionally, the first generator $G_{1}$ was regularized by the identity loss $L_{\left(id_{1}\right)}$ which means that the first generator network utilized the histopathologically stained H&E patch $\left(z_{target}\right)$ and reconstructed it at its output. We included only identity mapping loss $L_{\left(id_{1}\right)}$ for generator $G_{1}$ as we were interested in creating flawless H&E images. However, identity mapping loss $L_{\left(id_{2}\right)}$ for generator $G_{2}$ can be included in future studies when better reconstruction on NLM images is desired. Furthermore, each generator had its own discriminator model, which predicted the plausibility of the generated outputs. This is like the CGAN model explained earlier where each generator-discriminator pair was trained in an adversarial process. The architecture of the two discriminator models in the cycle CGAN model was similar to the Pix2Pix model; however, the architecture of generator networks was different.

The generator networks were inspired by the architecture proposed by Isola 2017 [14]. Both generator networks $\left(G_{1},G_{2}\right)$ used input image size 256×256, and the outputs were a computationally stained H&E patch $\left(z_{generated}\right)$ and a reconstructed NLM patch $\left({\tilde{x}}_{m}\right)$, respectively. The generator networks comprised of downsampling convolution blocks to encode the input, a sequence of six ResNet blocks, and upsampling convolution blocks that decodes the bottleneck features to an output. The shorthand notation of the generator network can be given as C7s1-64, D128, D256, R256, R256, R256, R256, R256, R256, U128, U64, C7s1-3 where C7s1-k denotes a 7×7 Convolution-InstanceNorm-ReLU layer with k filters and stride 1. Dk denotes a 3×3 Convolution-InstanceNorm-ReLU layer with k filters and stride 2. Uk denotes a 3×3 fractional-strided-Convolution-InstanceNorm-ReLU layer with k filters and stride 1/2. Rk denotes a ResNet block that contains two 3×3 convolutional layers with the same number of filters on both the layers. Like the CGAN model, the last layer of the generator network comprised of the tanh activation function. The weights of the generator networks were updated through adversarial loss, identity loss [29] and cycle consistency losses [29] (including forward and backward cycle). Mathematically, the full objective function of the cycle CGAN model can be given as

(3) $$\begin{array}{cc} L(G_{1},G_{2},D_{1},D_{2}) & =L_{\left(D_{1}\right)}(G_{1},D_{1},X,Y)+L_{\left(id_{1}\right)}(G_{1},D_{1},X)\\ & +\lambda L_{\left(cyc_{f}\right)}(G_{1},G_{2})+\lambda L_{\left(cyc_{b}\right)}(G_{1},G_{2}), \end{array}$$

where $L_{\left(D_{1}\right)}$ is the adversarial loss through which the generator 1 was updated. This is mean squared error instead of binary cross-entropy in the Pix2Pix model, as it provided better results in the literature [14]. In future, the adversarial loss for second generator $L_{\left(D_{2}\right)}$ can also be added. The identity loss $L_{\left(id_{1}\right)}$ and the forward and backward cycle loss $L_{\left(cyc_{f}\right)}$ , $L_{\left(cyc_{b}\right)}$ are the mean absolute error. The four losses were weighted by a factor of 1, 5, 10 and 10, respectively. The training of each generator-discriminator pair was similar to the CGAN model.

### 2.6 Model training and removal of patch-effect

The Pix2Pix model and the cycle CGAN model were trained on patches obtained from the NLM images and histopathologically stained H&E images. Both models were trained for 100 epochs, and a batch size of one patch was used. The model training was performed using Python 3.5 on a commercially available PC system with NVIDIA GeForce GTX 1060, 6GB. The generator models were saved after every fifth epoch, and the model that generated clinically acceptable H&E images from the training dataset (on visual inspection) was used for predicting the images from the test dataset. During the testing phase, the test images were pre-processed in a similar fashion as the training dataset. Further, the prediction of the images in the test dataset was performed on patches, which were subsequently combined to a whole image. Combining the patches resulted into a “patch-effect” which was visible at the edge of each patch in the combined image, precisely the pixel at every 256^th^ row or column in the whole image. For this purpose, the pixels which showed the patch-effect were linearly interpolated with its neighbouring three pixels. The generated H&E images (before and after correction of patch-effect) from both Pix2Pix and cycle CGAN models were visually inspected (Fig. S1 in Supplement 1). In addition to the visual inspection, quantitative evaluation of the computationally stained H&E images obtained from both pseudo-stain H&E models was performed. The quantitative evaluation was based on three metrics explained further.

### 2.7 Evaluation method

For performance quantification, the mean squared error (MSE) was utilized to calculate the error between the histopathologically stained H&E image ($z_{target}$) and the computationally stained H&E image ($z_{generated}$) [37]. However, MSE has a limitation caused due to arbitrarily high numbers which are difficult to standardize. Also, the MSE metric is inconsistent with the human perception ability [37]. Therefore, two other metrics namely structure similarity index (SSIM) [37,38] and color shading similarity (CSS) [39], which are well-suited for evaluating GAN performances, were utilized.

The structure similarity index [37,38] quantifies the perceptual similarity between the two images ($z_{target},z_{generated}$) by considering the contrast, luminance and texture of these images. Mathematically, SSIM between two images X and Y can be given as

(4) $$\text{SSIM}(X,Y)=\frac{\left(2\mu_{X}\mu_{Y}+c_{1})(2\sigma_{XY}+c_{2}\right)}{\left(\mu_{X}^{2}+\mu_{Y}^{2}+c_{1})(\sigma_{X}^{2}+\sigma_{Y}^{2}+c_{2}\right)},$$

where μ is mean of an image, σ is the standard deviation of an image, $\sigma_{XY}$ is the cross-covariance of the two images and $C=(c_{1},c_{2})$ are constants to avoid division by zero. For a multichannel image or an RGB image, the SSIM metric is calculated for each channel separately and the average SSIM value is considered. Higher values of SSIM indicate higher structural similarity between the two images.

Another metric called color shading similarity (CSS) [39] was used to quantify the similarity in the colors of the pixels in the histopathologically stained H&E image ($z_{target}$) and the computationally stained H&E image ($z_{generated}$). The CSS is calculated by converting both images in the CIELAB space and utilizing only the color channels A* and B*. For each color channel, the mathematical formulation of the CSS metric between two images X and Y is given by

(5) $$\text{CSS}(X,Y)=\frac{1}{N}\sum_{i=1}^{N}Ind_{i}\cdot Sim(X_{i},Y_{i}),$$

where, $Sim(X_{i},Y_{i})$ is the similarity between pixel $X_{i}$ of the histopathologically stained H&E image and pixel $Y_{i}$ of the computationally stained H&E image, i is the index used for the N pixels in the image. Mathematically, Sim and Ind are given as,

(6) $$\begin{array}{c} Sim(X_{i},Y_{i})=1-\frac{dist(X_{i},Y_{i})}{\max(dist)},\\ Ind_{i}=\left\{ \begin{array}{cc} 1; & \text{if}\;Sim(X_{i},Y_{i})>\text{threshold},\\ 0; & \text{if}\;Sim(X_{i},Y_{i})\leq \text{threshold}. \end{array}\right. \end{array}$$

In our case, the threshold was set to 0.5, and dist was the absolute distance. Higher values of the CSS indicate a higher color similarity. Likewise, the three metrics were evaluated for all the computationally stained H&E images (excluding the background region) from the Pix2Pix and the cycle CGAN models. The average of the three metrics calculated for all the 19 images from the training and test dataset is reported in Table 1 and Fig. 7.

**Table 1.** The average of the three evaluation metrics obtained for the 19 images using the Pix2Pix model and the cycle CGAN model are given for training and testing dataset. For reference purpose, the three metrics were also calculated with the same histopathologically stained H&E image. It is seen that MSE values are very large for both models, whereas SSIM and CSS are almost similar for both models. This means that the pixel values of computationally stained H&E images are different, but the overall structural and color information is acceptable. Furthermore, the metric values for training and testing dataset do not have a large difference, which indicates that the models are minimally overfitted.

	MSE	SSIM	CSS
	Training dataset		
Pathological stained H&E image	0.00	1.00	1.00
Pix2Pix stained H&E image	4.69$\times 10^{3}$	0.52	0.93
Cycle CGAN stained H&E image	10.26$\times 10^{3}$	0.49	0.91
	Testing dataset		
Pathological stained H&E image	0.00	1.00	1.00
Pix2Pix stained H&E image	4.27$\times 10^{3}$	0.60	0.94
Cycle CGAN stained H&E image	7.79$\times 10^{3}$	0.59	0.93

## 3. Results

The training of the Pix2Pix model for 100 epochs required ∼7 hours, while the cycle CGAN model required more than ∼100 hours on our commercial PC. The training of the cycle CGAN model was terminated after 60 epochs as no significant improvement in the computationally stained H&E patches was observed visually. Furthermore, it was observed that the generator and discriminator losses for the Pix2Pix model and the cycle CGAN model fluctuated throughout the training process. The discriminator loss for both models had difficulties to remain converged at an ideal value ≥0.5, which can be due to high variance [40,41] and noise in our dataset. The computationally stained H&E patches obtained using the saved generator models were visually assessed for their quality. A detailed explanation of the assessment procedure is provided in the next section.

### 3.1 Visual similarity of the GAN generated images

Computational staining of NLM images using CGANs achieved visually pleasing results compared to the state-of-the-art machine learning model used by Bocklitz et al. in 2016 [12] (see Fig. 1). As the visual appearance directly impacts the histopathological examination of any disease [25,42], its qualitative evaluation is vital. For this purpose, computationally stained H&E patches using the Pix2Pix and the cycle CGAN model from the training and testing dataset were inspected for different tissue regions.

**Fig. 4.**
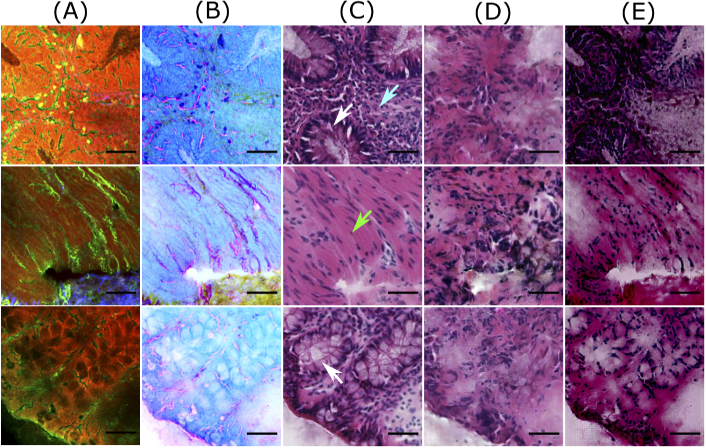
Bad quality predictions of patches from the training dataset. Columns (A) and (B) visualize NLM patches and contrast inverted NLM patches, column (C) shows histopathologically stained H&E patch, and columns (D) and (E) visualize computationally stained H&E patch generated by the Pix2Pix model and the cycle CGAN model, respectively. The scale bar represents 50 μm. Here the patches generated by the cycle CGAN model show a promising translation of corresponding NLM patches; however, the colors in stroma regions (marked by green arrows) and nuclei signals (marked by cyan arrows) are not well represented. The computationally stained H&E patches generated by the Pix2Pix model have a low spatial resolution, as the structures within the crypts (pointed by white arrows) are not visible.

**Fig. 5.**
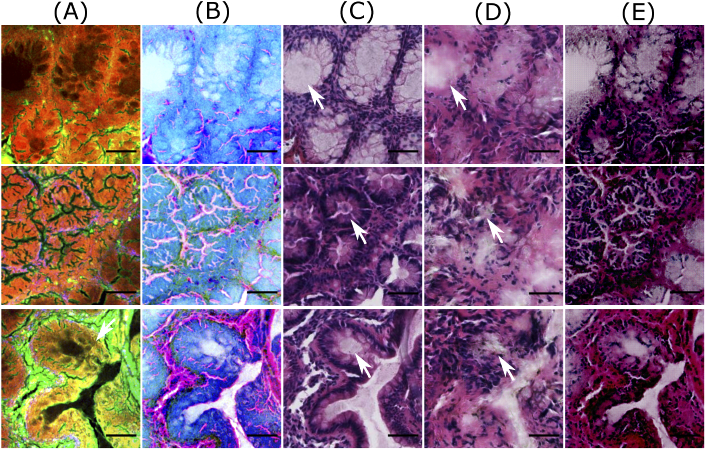
Good quality predictions of patches from the test dataset. Column (A) shows NLM patches, column (B) visualizes contrast inverted NLM patches, column (C) shows histopathologically stained H&E patch, and columns (D) and (E) depict computationally stained H&E patch by the Pix2Pix model and the cycle CGAN model, respectively. The scale bar represents 50 μm. Here, the computationally stained H&E patches by the cycle CGAN model shows a good quality translation of NLM patches, whereas the translation by the Pix2Pix model produces blurry results within the crypt regions (marked by white arrows).

**Fig. 6.**
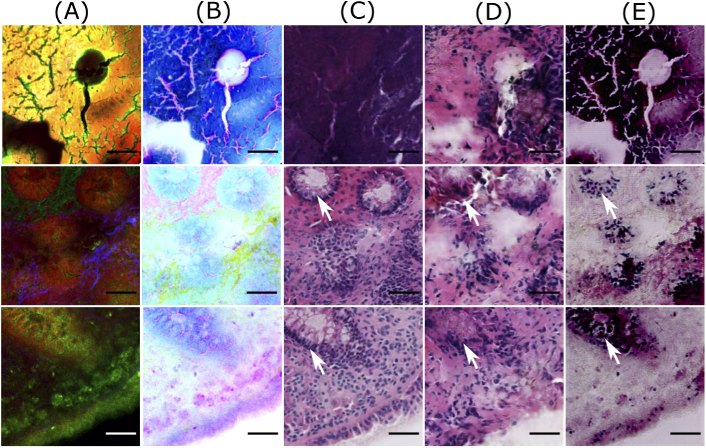
Bad quality predictions of patches from the test dataset. Column (A) shows NLM patches, column (B) visualizes contrast inverted NLM patches, column (C) shows histopathologically stained H&E patch, and columns (D) and (E) depict computationally stained H&E patch by the Pix2Pix model and the cycle CGAN model, respectively. The scale bar represents 50 μm. Here, computationally stained H&E patches (columns D and E) are not similar to histopathologically stained H&E patches (column C), as histopathologically stained H&E patches show different structures than the corresponding NLM patch. Furthermore, in dark NLM patches (second and third row), the computationally stained H&E patches fail to generate appropriate color contrast in crypt regions (marked by white arrows).

Figure 3 and Fig. 4 show computationally stained H&E patches in good and bad quality from the training dataset, respectively. Similarly, Fig. 5 and Fig. 6 show good and bad quality computationally stained H&E patches from the testing dataset. For the good quality patches, it can be observed that computationally stained H&E patches in columns D and E of Fig. 3 and Fig. 5 look visually similar to the histopathologically stained H&E patches in column C. Precisely, the good computationally stained H&E patches show a color contrast similar to the histopathologically stained H&E patches, i.e. the region within the crypts (marked by white arrows) is light or pale pink, whereas the epithelial layer or mucosa region outlining the crypts appears dark purple or dark pink. Furthermore, the regions showing nuclei (marked by cyan arrow) are generated better by the Pix2Pix model compared to the cycle CGAN model. Overall, the tissue structures in the good computationally stained H&E patches are clinically acceptable. Nevertheless, the bad quality patches as shown in columns D and E of Fig. 4 and Fig. 6 looks visually different than the histopathologically stained H&E patches shown in column C. In the bad computationally stained H&E patches, the structures within the crypts (marked by white arrows) are lost and the colors in stroma regions (marked with green arrow) are wrongly modelled. Furthermore, the nuclei signals present in Fig. 4, column C (marked with cyan arrow) are not efficiently generated in column D and E. Inconsistent modelling of nuclei signals in computationally stained H&E images is expected as NLM images show negative contrast for the cell nucleus. Sometimes the nuclei are out of focus in NLM images and due to this fact, there is no nuclei contrast. It is observed that nuclei signals and stroma region are occasionally generated in column C which can be a systematic error of GAN based model. Nevertheless, in our application (in contrast to oncology) crypt structures are more important rather than the shape of the cell nuclei in the stroma.

The variations between the NLM and its corresponding histopathologically stained H&E patch is due to the optical properties of the NLM imaging technique. The NLM imaging technique shows structures in a focal plane within the tissue section, which is approximately ∼5μm. In contrast, the histopathological staining technique reveals the structures from the entire thickness of the tissue section (∼20μm). Thus, both modalities show slightly different structures, which can be seen through a fine observation of patches in columns A and C of Figs. 3–6. This is the reason that an exact correspondence between computationally stained H&E patch and histopathologically stained H&E patch cannot be achieved for all images in the dataset.

In addition to the structural differences between histopathologically stained H&E patches and computationally stained H&E patches, there are other critical issues which need attention. Foremost, it can be seen from Figs. 3–6 that the Pix2Pix model generates low spatial resolution images as compared to images generated using the cycle CGAN model. The Pix2Pix model tends to lose detailed boundaries and edges within the crypt region (marked by white arrows) and show a blurry effect, at least for images from the test dataset (see Fig. 5 and Fig. 6). One of the reasons for the loss of detailed information can be the mean absolute error which was used as the objective function while training the generator network in the Pix2Pix model. The next critical issue was that the computationally stained H&E patches generated by the cycle CGAN model showed higher color contrast, thus making the colors more vivid. The high color contrast in the computationally stained H&E patch by the cycle CGAN model can be due to unsupervised training. It is suspected that the unsupervised training of the cycle CGAN model can be sensitive to alterations in the pixel intensity of the NLM images, staining inconsistencies in the histopathologically stained H&E image or a pre-processing effect [40,41]. Nevertheless, the problem of high color contrast observed in the computationally stained H&E patches from the cycle CGAN model can be reduced by simple image processing methods like contrast adjustment [43]. Overall, from the visual appearance of the computationally stained H&E patches, it can be seen that an exact correspondence with histopathologically stained H&E patches cannot be achieved. However, the computationally stained H&E patches in column D and E of Figs. 3–6 generated using the Pix2Pix and the cycle CGAN model provide an acceptable translation of NLM patches. In Fig. S2 and Fig. S3 in Supplement 1, computationally stained H&E patches combined into an image, and computationally stained H&E images from the method presented in [12] are shown. Furthermore, the computationally stained H&E image from the cycle CGAN model followed by contrast reduction with a factor of 0.7 is also visualized. The computationally stained H&E images were also examined by a histologist for its clinical significance. According to the expert analysis, both models show promising results for translating NLM images. In addition to visual analysis, a quantitative evaluation was done and is discussed below.

### 3.2 Quality of computationally staining based on metrics

An evaluation of the Pix2Pix and the cycle CGAN model was performed based on three metrics: MSE, SSIM and CSS. The average values of MSE, SSIM and CSS for training and testing dataset are reported in Table 1. Here, the three metrics were calculated with the same histopathologically stained H&E image and were considered as baseline values. The aim of the pseudo-stain H&E models was to acquire values “close” to these baseline values.

From Table 1, it can be seen that the computationally stained H&E images generated from both models show very high MSE and low SSIM values as compared to the baseline values. High MSE and low SSIM values were expected as an exact correspondence of computationally stained H&E image with its histopathologically stained H&E image cannot be achieved. Thus, the interpretation of the image quality based on the MSE and SSIM metric is unfair. Despite the high MSE or low SSIM values, the computationally stained H&E images from both models shown in Fig. S2 and Fig. S3 in Supplement 1 have promising visual appearance when compared to its NLM image. Furthermore, the MSE values are higher for the cycle CGAN model as compared to the Pix2Pix model. Higher MSE values using the cycle CGAN model are suspected due to largely different pixel values of computationally stained H&E patches. On the other hand, the SSIM and CSS metrics report similar performance for the Pix2Pix and the cycle CGAN model, which implies that the overall structural and color content of the computationally stained H&E image is acceptable. Furthermore, the metric values are similar for training and testing dataset (see Table 1) which shows that the models are minimally overfitted. The mean SSIM and mean CSS metric for the training and the testing dataset using both models are >0.50 and >0.90, respectively. The three metrics for all images are given in Table S1 in Supplement 1.

**Fig. 7.**
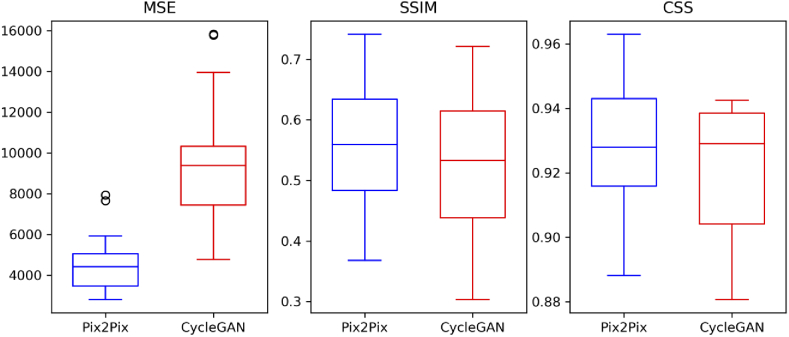
The boxplot shows a quantitative comparison of the Pix2Pix and the cycle CGAN model based on the three evaluation metrics. The MSE metric is higher for the cycle CGAN model and shows larger variation. This is expected as the pixel values of computationally stained H&E images generated by the cycle CGAN model differ more than the computationally stained H&E images generated by the Pix2Pix model. Nevertheless, the CSS and SSIM metric is in a similar range for both models, which implies that the content of computationally stained H&E images generated by both models is similar.

In addition to Table S1 in Supplement 1, the range of the three metrics is given in Fig. 7, which shows a large variance in the three metrics. The large variance in the three metrics was expected and can possibly be due to the large variance in the dataset. Nevertheless, the color information produced by both pseudo-stain H&E models is close to the baseline value (1.0). The three metrics for a randomly chosen H&E image generated using both models are given in Fig. S2 for the testing dataset and Fig. S3 for the training dataset in Supplement 1. Lastly, there was no significant difference in the performance metrics before and after correction of patch-effect of computationally stained H&E images.

## 4. Discussion

The computationally stained H&E images generated by the supervised (Pix2Pix) and the unsupervised (cycle CGAN) pseudo-stain H&E model showed a substantial improvement to the state-of-the-art machine learning model [12] based on color contrast. However, it was observed that the training time of the machine learning model was less (∼4 hours) than the training time of CGAN models presented here. Nevertheless, we believe that the cycle CGAN model can provide better computationally stained H&E images. Furthermore, pseudo-stain H&E model can be applied for multi-modality conversion, augment the NLM images and remove noise from multimodal images. In all these tasks, a systematic investigation is needed. Realization of these tasks using the cycle CGAN model can cause staining protocols less labor intensive [11]. However, there are some important aspects considered for training both models, particularly, the training dataset, the pre-processing of the histopathologically stained H&E image and NLM image and the objective function. These aspects are discussed in more detail below.

### 4.1 Effect of training dataset

The first aspect is the training dataset utilized for constructing the pseudo-stain H&E models. Similar to any other deep learning networks, pseudo-stain H&E model based on CGANs are also sensitive to the training dataset. It was observed that the training dataset with a large number of noisy patches or background patches affected the convergence of the generator and the discriminator network. Therefore, patch filtering was vital. Furthermore, a large number of trainable parameters in the generator and the discriminator network can easily cause overfitting on the training dataset. This was a major problem in the supervised approach, e.g. the Pix2Pix model, where the target patches were available. The overfitting on training dataset is seen in Fig. 5 and Fig. 6, column D. Here, we see the patches from the test dataset lose their spatial resolution compared to the patches from the training dataset in the Fig. 3.

Contrarily, in the unsupervised approach, the cycle CGAN model trained on unpaired image data required a quality check of the training dataset. It was observed that for the cycle CGAN model, the color of the computationally stained H&E patches was influenced by the color of the majority of patches in the training dataset. For instance, the cycle CGAN model trained with a large number of patches from the stroma region, i.e. patches with pink color, was likely to produce pinkish H&E images. Therefore, to create a balance in the color of the generated H&E images, a manual quality check of the patches in the training dataset was crucial for the training of the cycle CGAN model. We believe with an increasing dataset and computation power, the performance of both pseudo-stain H&E models can be improved.

### 4.2 Effect of the objective function and performance metric

The next aspect for training the pseudo-stain H&E models is the objective function and the performance metric. We begin with the selection of the objective function. Foremost, an appropriate selection of the objective function for the generator and the discriminator network is important to generate clinically acceptable H&E images. In this regard, researchers have shown the benefits of using various objective functions like the style transfer loss [44], the perceptual loss [44], the total variation loss [24] and the image gradient loss [45]. Nevertheless, in our case, the L1-loss for the generator network of the cycle CGAN model showed acceptable results. We believe that the addition of other losses to the objective function can improve the perceptual quality of the generated H&E images yet increasing the model complexity. These losses can be applied for the Pix2Pix and the cycle CGAN model and researched in future studies.

The second aspect is the performance metric. The performance metrics used in this work were calculated on the pixel basis and are sensitive to slight variations in the computational H&E images. For instance, a histopathologically stained H&E image and a computationally stained H&E image offset by one pixel can create a major difference in these performance metrics [44]. This problem is often encountered during registration of H&E image and NLM image. Therefore, the high values of the MSE and low values of the SSIM metric shown in Table 1 is justified. In future studies, an objective function that can evaluate the global quality of the computationally stained H&E images can be utilized.

### 4.3 Effect of image normalization and contrast inversion

In the end, this section discusses the aspect of image normalization and “contrast-inversion” performed while training the pseudo-stain H&E models. Foremost, the normalization methods of both NLM and histopathologically stained H&E images was essential to avoid multiplications of large numbers during the training process. During the training phase, several methods of normalizing the NLM images and histopathologically stained H&E images were evaluated. It was observed that the NLM and histopathologically stained H&E patches scaled in the range [−1,1] generated the best results. It was also observed that scaling of NLM and histopathologically stained H&E images instead of scaling its patches did not affect the training or model performance. Furthermore, scaling the NLM and/or histopathologically stained H&E patches in the range [0,1] led to the failure of the discriminator network by immediately converging the discriminator losses to zero. The scaling of histopathologically stained H&E patches was essential due to the tanh activation function used in the last layer of the generator network [13]. These findings coincide with the results of Ref. [46].

In addition to the normalization, “contrast-inversion” of the NLM images was performed to remove the “inverse-color” effect [47]. This effect was seen when the original NLM image (without contrast inversion) was used (see Fig. S4 in Supplement 1). This effect was especially seen in the unsupervised approach, i.e. using the cycle CGAN model. Because of this effect, the crypt region was transformed into dark purple instead of light pink and vice versa. Therefore, “contrast-inversion” was an important step for modality conversion, especially where the two modalities showed significantly different color contrasts.

## 5. Conclusion

Computational staining of NLM images is beneficial from a clinical perspective as it prevents the staining procedures and reduces manual effort. This work was an improvement of the state-of-the-art method, which utilized the conventional machine learning approach for computational staining of NLM images. On the contrary, this work presented a supervised and unsupervised approach to computationally stain NLM images into H&E stained images. The supervised approach utilized the Pix2Pix model, and the unsupervised approach used the cycle CGAN model. For the Pix2Pix model, a corresponding pair of NLM image and histopathologically stained H&E image was required. Therefore, image registration of the histopathologically stained H&E image was crucial. On the other hand, the cycle CGAN model did not require the corresponding pair of the NLM image and histopathologically stained H&E image. Thus, the effort of image registration and pathological staining was reduced. The qualitative and quantitative evaluation of both models showed comparable results using evaluation metrics based on color, texture and perceptual quality. The evaluation metric like mean squared error reported values >5$\times 10^{3}$ and >8$\times 10^{3}$ for the Pix2Pix and the cycle CGAN model, respectively. In contrast, the evaluation metric, including SSIM and CSS reported values >0.50 and >0.90 for both models, respectively. In addition to quantitative evaluation, various pre- and post-processing procedures were explored in this work, however more advanced post-processing procedures could be investigated in future. Furthermore, a cycle CGAN model that can perform multiple staining using a NLM image can be one of the future research directions. The cycle CGAN model can also be investigated for additional benefits like the artificial generation of NLM images, increasing the spatial resolution of the computationally stained H&E images and removing fluorescence effect from the reconstructed NLM images. Overall, the results showed several benefits of using computational staining of NLM images than performing histopathological staining in laboratories. Thus, the computational staining approach should be encouraged in clinics to benefit the pathological and clinical field of science.

## Code availabilty

https://github.com/Bocklitz-Lab/Pseudo_HE_modelling.git

## Supplemental document

See Supplement 1 for supporting content.

## References

[1] Bocklitz T. Silge A. Bae H. Rodewald M. Legesse F. B. Meyer T. Popp J., “Non-invasive imaging techniques: From histology to in vivo imaging,” in *Molecular Imaging in Oncology* (Springer, 2020), pp. 795–812. 10.1007/978-3-030-42618-7_25 32594407

[2] Vogler N. Heuke S. Bocklitz T. W. Schmitt M. Popp J., “Multimodal imaging spectroscopy of tissue,” Annu. Rev. Anal. Chem. 8(1), 359–387 (2015). 10.1146/annurev-anchem-071114-040352 26070717

[3] Cicchi R. Pavone F. S., “Multimodal nonlinear microscopy: A powerful label-free method for supporting standard diagnostics on biological tissues,” J. Innovative Opt. Health Sci. 07(05), 1330008 (2014). 10.1142/S1793545813300085

[4] Heuke S. Vogler N. Meyer T. Akimov D. Kluschke F. Röwert-Huber H. Lademann J. Dietzek B. Popp J., “Multimodal mapping of human skin,” Br. J. Dermatol. 169(4), 794–803 (2013). 10.1111/bjd.12427 23927013

[5] Heuke S. Vogler N. Meyer T. Akimov D. Kluschke F. Röwert-Huber H.-J. Lademann J. Dietzek B. Popp J., “Detection and discrimination of non-melanoma skin cancer by multimodal imaging,” in *Healthcare*, vol. 1(1) (Multidisciplinary Digital Publishing Institute, 2013), pp. 64–83. 27429131 10.3390/healthcare1010064 PMC4934506

[6] Guo S. Pfeifenbring S. Meyer T. Ernst G. von Eggeling F. Maio V. Massi D. Cicchi R. Pavone F. S. Popp J. Bocklitz T., “Multimodal image analysis in tissue diagnostics for skin melanoma,” J. Chemom. 32(1), e2963 (2018). 10.1002/cem.2963

[7] Heuke S. Chernavskaia O. Bocklitz T. Legesse F. B. Meyer T. Akimov D. Dirsch O. Ernst G. von Eggeling F. Petersen I. Guntinas-Lichius O. Schmitt M. Popp J., “Multimodal nonlinear microscopy of head and neck carcinoma – toward surgery assisting frozen section analysis,” Head Neck 38(10), 1545–1552 (2016). 10.1002/hed.24477 27098552

[8] Meyer T. Guntinas-Lichius O. von Eggeling F. Ernst G. Akimov D. Schmitt M. Dietzek B. Popp J., “Multimodal nonlinear microscopic investigations on head and neck squamous cell carcinoma: Toward intraoperative imaging,” Head Neck 35(9), E280–E287 (2013). 10.1002/hed.23139 22987435

[9] Meyer T. Bergner N. Krafft C. Akimov D. Dietzek B. Popp J. Bielecki C. Romeike B. F. Reichart R. Kalff R., “Nonlinear microscopy, infrared, and Raman microspectroscopy for brain tumor analysis,” J. Biomed. Opt. 16(2), 021113 (2011). 10.1117/1.3533268 21361676

[10] Schürmann S. Foersch S. Atreya R. Neumann H. Friedrich O. Neurath M. F. Waldner M. J., “Label-free imaging of inflammatory bowel disease using multiphoton microscopy,” Gastroenterology 145(3), 514–516 (2013). 10.1053/j.gastro.2013.06.054 23850960

[11] Orringer D. A. Pandian B. Niknafs Y. S. Hollon T. C. Boyle J. Lewis S. Garrard M. Hervey-Jumper S. L. Garton H. J. L. Maher C. O. Heth J. A. Sagher O. Wilkinson D. A. Snuderl M. Venneti S. Ramkissoon S. H. McFadden K. A. Fisher-Hubbard A. Lieberman A. P. Johnson T. D. Xie X. S. Trautman J. K. Freudiger C. W. Camelo-Piragua S., “Rapid intraoperative histology of unprocessed surgical specimens via fibre-laser-based stimulated Raman scattering microscopy,” Nat. Biomed. Eng. 1(2), 0027 (2017). 10.1038/s41551-016-0027 28955599 PMC5612414

[12] Bocklitz T. W. Salah F. S. Vogler N. Heuke S. Chernavskaia O. Schmidt C. Waldner M. J. Greten F. R. Bräuer R. Schmitt M., “Pseudo-HE images derived from CARS/TPEF/SHG multimodal imaging in combination with Raman-spectroscopy as a pathological screening tool,” BMC Cancer 16(1), 534 (2016). 10.1186/s12885-016-2520-x 27460472 PMC4962450

[13] Goodfellow I. J., “NIPS 2016 tutorial: Generative adversarial networks,” ArXiv **abs/1701.00160**, (2017).

[14] Isola P. Zhu J.-Y. Zhou T. Efros A. A., “Image-to-image translation with conditional adversarial networks,” in Proceedings of the IEEE conference on computer vision and pattern recognition, (2017), pp. 1125–1134.

[15] Zhu J.-Y. Park T. Isola P. Efros A. A., “Unpaired image-to-image translation using cycle-consistent adversarial networks,” in Proceedings of the IEEE international conference on computer vision, (2017), pp. 2223–2232.

[16] Mirza M. Osindero S., “Conditional generative adversarial nets,” arXiv preprint arXiv:1411.1784 (2014).

[17] Ma Y. Chen X. Zhu W. Cheng X. Xiang D. Shi F., “Speckle noise reduction in optical coherence tomography images based on edge-sensitive cGAN,” Biomed. Opt. Express 9(11), 5129–5146 (2018). 10.1364/BOE.9.005129 30460118 PMC6238896

[18] Zheng R. Liu L. Zhang S. Zheng C. Bunyak F. Xu R. Li B. Sun M., “Detection of exudates in fundus photographs with imbalanced learning using conditional generative adversarial network,” Biomed. Opt. Express 9(10), 4863–4878 (2018). 10.1364/BOE.9.004863 30319908 PMC6179403

[19] Zhang C. Wang K. An Y. He K. Tong T. Tian J., “Improved generative adversarial networks using the total gradient loss for the resolution enhancement of fluorescence images,” Biomed. Opt. Express 10(9), 4742–4756 (2019). 10.1364/BOE.10.004742 31565522 PMC6757480

[20] Zhang H. Fang C. Xie X. Yang Y. Mei W. Jin D. Fei P., “High-throughput, high-resolution deep learning microscopy based on registration-free generative adversarial network,” Biomed. Opt. Express 10(3), 1044–1063 (2019). 10.1364/BOE.10.001044 30891329 PMC6420277

[21] Ouyang J. Mathai T. S. Lathrop K. Galeotti J., “Accurate tissue interface segmentation via adversarial pre-segmentation of anterior segment OCT images,” Biomed. Opt. Express 10(10), 5291–5324 (2019). 10.1364/BOE.10.005291 31646047 PMC6788614

[22] Jiang H. Chen X. Shi F. Ma Y. Xiang D. Ye L. Su J. Li Z. Chen Q. Hua Y. Xu X. Zhu W. Fan Y., “Improved cGAN based linear lesion segmentation in high myopia ICGA images,” Biomed. Opt. Express 10(5), 2355–2366 (2019). 10.1364/BOE.10.002355 31149376 PMC6524580

[23] Halupka K. J. Antony B. J. Lee M. H. Lucy K. A. Rai R. S. Ishikawa H. Wollstein G. Schuman J. S. Garnavi R., “Retinal optical coherence tomography image enhancement via deep learning,” Biomed. Opt. Express 9(12), 6205–6221 (2018). 10.1364/BOE.9.006205 31065423 PMC6490980

[24] Rivenson Y. Wang H. Wei Z. Zhang Y. Gunaydin H. Ozcan A., “Deep learning-based virtual histology staining using auto-fluorescence of label-free tissue,” Nat. Biomed. Eng. 3, 466–477 (2018). 10.1038/s41551-019-0362-y

[25] Bayramoglu N. Kaakinen M. Eklund L. Heikkila J., “Towards virtual H&E staining of hyperspectral lung histology images using conditional generative adversarial networks,” in Proceedings of the IEEE International Conference on Computer Vision Workshops, (2017), pp. 64–71.

[26] Liu T. Wei Z. Rivenson Y. de Haan K. Zhang Y. Wu Y. Ozcan A., “Deep learning-based color holographic microscopy,” J. Biophotonics 12(11), e201900107 (2019). 10.1002/jbio.201900107 31309728

[27] Nehme E. Weiss L. E. Michaeli T. Shechtman Y., “Deep-storm: super-resolution single-molecule microscopy by deep learning,” Optica 5(4), 458–464 (2018). 10.1364/OPTICA.5.000458

[28] Wang H. Rivenson Y. Jin Y. Wei Z. Gao R. Günaydin H. Bentolila L. A. Kural C. Ozcan A., “Cross-modality deep learning achieves super-resolution in fluorescence microscopy,” in 2019 Conference on Lasers and Electro-Optics (CLEO) (IEEE, 2019), pp. 1–2.

[29] Xu Z. Moro C. F. Bozóky B. Zhang Q., “GAN-based virtual re-staining: a promising solution for whole slide image analysis,” arXiv preprint arXiv:1901.04059 (2019).

[30] Chernavskaia O. Heuke S. Vieth M. Friedrich O. Schürmann S. Atreya R. Stallmach A. Neurath M. F. Waldner M. Petersen I., “Beyond endoscopic assessment in inflammatory bowel disease: real-time histology of disease activity by non-linear multimodal imaging,” Sci. Rep. 6(1), 29239 (2016). 10.1038/srep29239 27406831 PMC4942779

[31] Roche A. Malandain G. Pennec X. Ayache N., “The correlation ratio as a new similarity measure for multimodal image registration,” in International Conference on Medical Image Computing and Computer-Assisted Intervention, (Springer, 1998), pp. 1115–1124.

[32] Pradhan P. Meyer T. Vieth M. Stallmach A. Waldner M. Schmitt M. Popp J. Bocklitz T., “Semantic segmentation of non-linear multimodal images for disease grading of inflammatory bowel disease: A segnet-based application,” in International Conference on Pattern Recognition Applications and Methods 2019, (2019).

[33] Pradhan P. Guo S. Ryabchykov O. Popp J. Bocklitz T. W., “Deep learning a boon for biophotonics?” J. Biophotonics 13, e201960186 (2020). 10.1002/jbio.201960186 32167235

[34] Ronneberger O. Fischer P. Brox T., “U-net: Convolutional networks for biomedical image segmentation,” in International Conference on Medical image computing and computer-assisted intervention, (Springer, 2015), pp. 234–241.

[35] Cho H. Lim S. Choi G. Min H., “Neural stain-style transfer learning using GAN for histopathological images,” arXiv preprint arXiv:1710.08543 (2017).

[36] Kingma D. P. Ba J., “Adam: A method for stochastic optimization,” arXiv preprint arXiv:1412.6980 (2014).

[37] Ren H. Li J. Gao N., “Automatic sketch colorization with tandem conditional adversarial networks,” in 2018 11th International Symposium on Computational Intelligence and Design (ISCID), vol. 1 (IEEE, 2018), pp. 11–15.

[38] Wang Z. Simoncelli E. P., “Translation insensitive image similarity in complex wavelet domain,” in Proceedings.(ICASSP’05). IEEE International Conference on Acoustics, Speech, and Signal Processing, 2005., vol. 2 (IEEE, 2005), pp. ii/573–ii/576 Vol. 2.

[39] Wang D. Shi L. Wang Y. J. Man G. C. Heng P. A. Griffith J. F. Ahuja A. T., “Color quantification for evaluation of stained tissues,” Cytometry, Part A 79A(4), 311–316 (2011). 10.1002/cyto.a.21037 21387544

[40] McCann M. T. Ozolek J. A. Castro C. A. Parvin B. Kovacevic J., “Automated histology analysis: Opportunities for signal processing,” IEEE Signal Process. Mag. 32(1), 78–87 (2015). 10.1109/MSP.2014.2346443

[41] Bayramoglu N. Kannala J. Heikkilä J., “Deep learning for magnification independent breast cancer histopathology image classification,” in 2016 23rd International conference on pattern recognition (ICPR), (IEEE, 2016), pp. 2440–2445.

[42] BenTaieb A. Hamarneh G., “Adversarial stain transfer for histopathology image analysis,” IEEE Trans. Med. Imaging 37(3), 792–802 (2018). 10.1109/TMI.2017.2781228 29533895

[43] Gonzalez R. C. Woods R. E. Eddins S. L., *Digital Image Processing Using MATLAB* (Prentice-Hall, Inc., USA, 2003).

[44] Johnson J. Alahi A. Fei-Fei L., “Perceptual losses for real-time style transfer and super-resolution,” in European conference on computer vision (Springer, 2016), pp. 694–711.

[45] Nie D. Trullo R. Lian J. Petitjean C. Ruan S. Wang Q. Shen D., “Medical image synthesis with context-aware generative adversarial networks,” in International Conference on Medical Image Computing and Computer-Assisted Intervention (Springer, 2017), pp. 417–425. 10.1007/978-3-319-66179-7_48 PMC6044459 30009283

[46] Radford A. Metz L. Chintala S., “Unsupervised representation learning with deep convolutional generative adversarial networks,” CoRR **abs/1511.06434** (2016).

[47] Wang T. Lin Y., “CycleGAN with better cycles,” (2018).

