# Supplementary material for: Computational tissue staining of non-linear multimodal imaging using supervised and unsupervised deep learning
Source: Biomed Opt Express. 2021 Mar 23;12(4):2280–98. doi: 10.1364/BOE.415962 (PMC8086483; doi:10.1364/BOE.415962)
Supplement: Supplementary file 1 [file boe-12-4-2280-s001.pdf]

Supplemental Document

Biomedical Optics EXPRESS

Computational tissue staining of non-linear multimodal imaging using supervised and unsupervised deep learning: supplement

PRANITA PRADHAN,[1,2] TOBIAS MEYER,[2] MICHAEL VIETH,[3] ANDREAS STALLMACH,[4] MAXIMILIAN WALDNER,[5,6] MICHAEL SCHMITT,[1] JUERGEN POPP,[1,2] AND THOMAS BOCKLITZ[1,2,*]

[1] *Institute of Physical Chemistry and Abbe Center of Photonics, Friedrich-Schiller-University, Jena, Germany*
[2] *Leibniz Institute of Photonic Technology, Member of Leibniz Health Technologies Jena, Germany*
[3] *Institute of Pathology, Klinikum Bayreuth, Bayreuth, Germany*
[4] *Department of Internal Medicine IV (Gastroenterology, Hepatology, and Infectious Diseases), Jena University Hospital, Jena, Germany*
[5] *Erlangen Graduate School in Advanced Optical Technologies (SAOT), Friedrich-Alexander University of Erlangen-Nuremberg, 91052 Erlangen, Germany*
[6] *Medical Department 1, Friedrich-Alexander University of Erlangen-Nuremberg, Erlangen, Germany*
[*] *thomas.bocklitz@uni-jena.de*

This supplement published with The Optical Society on 23 March 2021 by The Authors under the terms of the Creative Commons Attribution 4.0 License in the format provided by the authors and unedited. Further distribution of this work must maintain attribution to the author(s) and the published article's title, journal citation, and DOI.

Supplement DOI: https://doi.org/10.6084/m9.figshare.14046467

Parent Article DOI: https://doi.org/10.1364/BOE.415962

Computational tissue staining of non-linear multimodal imaging using supervised and unsupervised deep learning

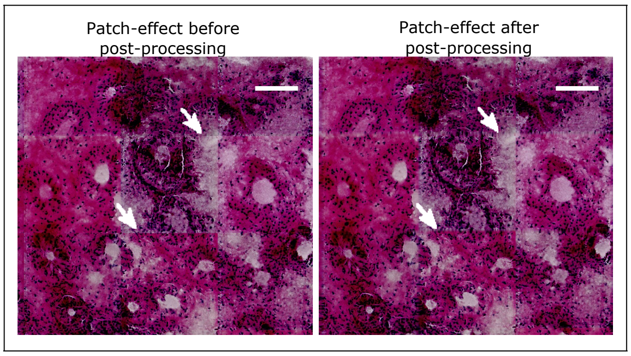

Fig. S1. This figure shows the effect of post-processing for removing 'patch-effect'. The 'patch-effect' was removed by interpolating pixel values of three neighbouring pixels at the end of every patch (256th pixel). This effect (shown in white arrows) was visible for few images and its removal did not significantly affect the performance metrics. The scale bar represents 100 μm

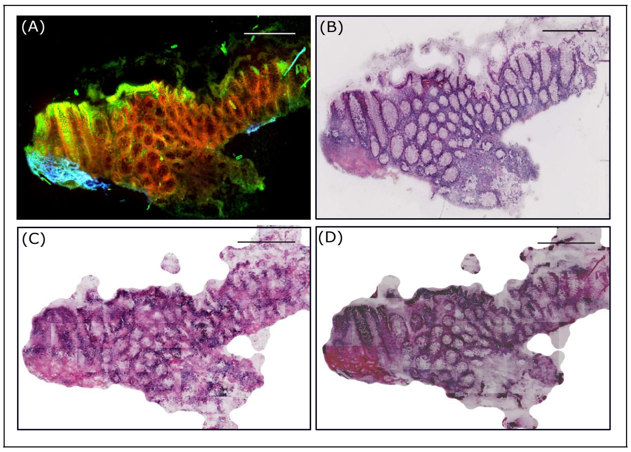

Fig. S2. (A) shows a non-linear multimodal image from test dataset, (B) visualizes corresponding histopathologically stained H&E image (unregistered), (C) shows the computational H&E image by the Pix2Pix (MSE = 4.4×10^3, SSIM = 0.65, CSS = 0.94) and (D) depicts computational H&E image by the cycle CGAN model (MSE = 8.4×10^3, SSIM = 0.63, CSS = 0.94). The contrast of the computational H&E image in (D) is reduced by a factor of 0.7. The images here are downsampled to 20% of original size for clarity. The scale bar represents 100 μm.

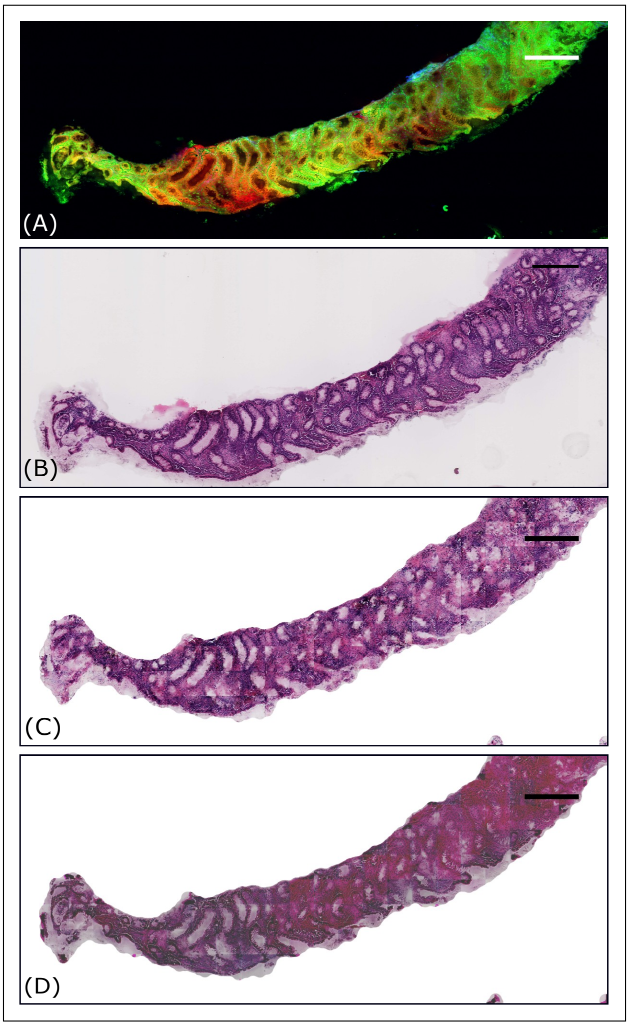

Fig. S3. (A) shows a non-linear multimodal image from training dataset, (B) visualizes corresponding histopathologically stained H&E image (unregistered), (C) shows the computational H&E image by the Pix2Pix (MSE = 2.8×10^3, SSIM = 0.74, CSS = 0.96) and (D) depicts computational H&E image by the cycle CGAN model (MSE = 5.9×10^3, SSIM = 0.72, CSS = 0.94). The contrast of the computational H&E image in (D) is reduced by a factor of 0.7. The images here are downsampled to 20% of original size for clarity. The scale bar represents 100 μm.

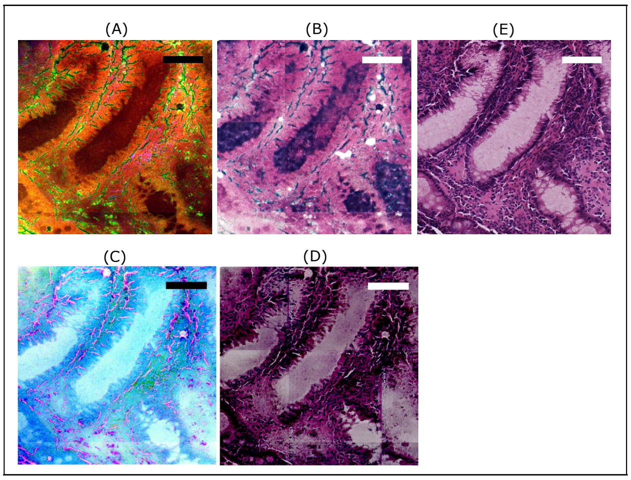

Fig. S4. This figure shows effect of 'contrast inversion' on the computational H&E images of the cycle CGAN model. (A) shows an original MM patch given to the cycle CGAN model and (B) visualizes the generated H&E patch. Here, we see that the colors in the generated H&E image are inverted when compared to the pathologically stained H&E patch shown in (E). Contrarily, (C) shows a contrast inverted MM patch and (D) depicts the generated H&E patch by the cycle CGAN model. Here, the color contrast in similar to the pathologically stained H&E patch shown in (E). The scale bar represents 100 μm.

Table S1. The quantitative metrics namely mean squared error (MSE), structure similarity index (SSIM) and color similarity index (CSS) evaluated for 19 images from the training and testing dataset is given for the Pix2Pix and the cycle CGAN models, respectively.

Image	Pix2Pix			Cycle CGAN		
	MSE	SSIM	CSS	MSE	SSIM	CSS
Train 1	3601.43	0.61	0.94	9373.09	0.57	0.92
Train 2	2800.83	0.74	0.96	5890.62	0.72	0.94
Train 3	4984.19	0.38	0.89	13955.34	0.33	0.89
Train 4	3337.39	0.56	0.93	8157.08	0.53	0.93
Train 5	4451.48	0.51	0.92	11509.74	0.47	0.90
Train 6	5100.78	0.49	0.92	9629.29	0.46	0.89
Train 7	4956.57	0.41	0.91	10389.17	0.38	0.90
Train 8	4167.94	0.52	0.92	8376.58	0.49	0.90
Train 9	7938.24	0.46	0.91	15823.56	0.41	0.91
Train 10	7657.41	0.39	0.88	9725.03	0.36	0.88
Train 11	3066.22	0.67	0.94	6749.37	0.65	0.93
Train 12	3057.30	0.65	0.94	8129.79	0.62	0.93
Train 13	5918.03	0.36	0.90	15783.79	0.30	0.90
Test 1	5617.82	0.60	0.92	6600.03	0.59	0.91
Test 2	3582.64	0.61	0.94	6633.59	0.60	0.94
Test 3	3827.09	0.65	0.94	4767.95	0.63	0.94
Test 4	3292.67	0.60	0.94	10118.23	0.58	0.93
Test 5	4416.22	0.64	0.94	8408.46	0.63	0.94
Test 6	4937.68	0.49	0.91	10265.39	0.48	0.93
